# Supplementary material for: Mechanisms governing the pioneering and redistribution capabilities of the non-classical pioneer PU.1
Source: Nat Commun. 2020 Jan 21;11:402. doi: 10.1038/s41467-019-13960-2 (PMC6972792; doi:10.1038/s41467-019-13960-2)
Supplement: Supplementary file 7 — Source data [file 41467_2019_13960_MOESM7_ESM.zip › Source_Data/Figure6/Figure6B_MotifScanOutput/ETS-specific/homerResults/motif7.similar.html]

motif7

## Information for motif7

G
A
C
T
A
G
C
T
A
T
C
G
A
G
C
T
A
C
G
T
C
G
T
A
A
C
G
T
A
G
T
C
C
G
A
T
  
Reverse Opposite:  

G
C
T
A
A
C
T
G
C
G
T
A
A
C
G
T
C
G
T
A
C
T
G
A
T
A
G
C
T
C
A
G
C
T
G
A
  

|  |  |
| --- | --- |
| p-value: | 1e-140 |
| log p-value: | -3.224e+02 |
| Information Content per bp: | 1.750 |
| Number of Target Sequences with motif | 1141.0 |
| Percentage of Target Sequences with motif | 14.91% |
| Number of Background Sequences with motif | 2383.0 |
| Percentage of Background Sequences with motif | 5.90% |
| Average Position of motif in Targets | 98.1 +/- 53.6bp |
| Average Position of motif in Background | 99.3 +/- 62.3bp |
| Strand Bias (log2 ratio + to - strand density) | 0.0 |
| Multiplicity (# of sites on avg that occur together) | 1.11 |
| Motif File: | file (matrix) reverse opposite |

### Similar de novo motifs found

|  |  |  |  |  |  |  |  |
| --- | --- | --- | --- | --- | --- | --- | --- |
| Rank | Match Score | Redundant Motif | P-value | log P-value | % of Targets | % of Background | Motif file |
| 1 | 0.972 | C T G A T A G C C G T A A T C G C G T A A C G T C G T A C T G A T C A G T G C A C G T A | 1e-134 | -309.379272 | 13.87% | 5.38% | motif file (matrix) |
| 2 | 0.918 | T A C G G C T A A C T G C G T A A C G T C G T A C G T A T C A G | 1e-130 | -301.215994 | 17.32% | 7.77% | motif file (matrix) |
| 3 | 0.956 | G C T A A C T G C G T A A C G T C G T A C G T A T C A G | 1e-128 | -295.737445 | 18.80% | 8.89% | motif file (matrix) |
| 4 | 0.967 | A G C T A G T C A C G T C G A T C G T A A C G T A G T C C G A T A G C T G A C T G C T A G A C T | 1e-122 | -282.315376 | 16.02% | 7.11% | motif file (matrix) |
| 5 | 0.949 | C A G T A G C T A G C T A C G T A C G T C G T A A C G T A G T C C G A T A T C G G A T C G A C T G A C T | 1e-100 | -232.060141 | 10.59% | 4.11% | motif file (matrix) |
